# Language and Traits of Autism Spectrum Conditions: Evidence of Limited Phenotypic and Etiological Overlap

**DOI:** 10.1002/ajmg.b.32262

**Published:** 2014-08-02

**Authors:** Mark J. Taylor, Tony Charman, Elise B. Robinson, Marianna E. Hayiou-Thomas, Francesca Happé, Philip S. Dale, Angelica Ronald

**Affiliations:** 1 Genes Environment Lifespan Laboratory, Centre for Brain and Cognitive Development, Department of Psychological Sciences, Birkbeck, University of London, London, UK; 2 King’s College London, Department of Psychology, Institute of Psychiatry, London, UK; 3 Analytic and Translational Genetics Unit, Massachusetts General Hospital, Boston, Massachusetts; 4 Department of Medicine, Harvard Medical School, Cambridge, Massachusetts; 5 Department of Psychology, University of York, York, UK; 6 King’s College London, MRC Social, Genetic and Developmental Psychiatry Centre, Institute of Psychiatry, De Crespigny Park, London, UK; 7 Department of Speech & Hearing Sciences, University of New Mexico, Albuquerque, New Mexico

**Keywords:** autism, receptive language, twin study

## Abstract

Language difficulties have historically been viewed as integral to autism spectrum conditions (ASC), leading molecular genetic studies to consider whether ASC and language difficulties have overlapping genetic bases. The extent of genetic, and also environmental, overlap between ASC and language is, however, unclear. We hence conducted a twin study of the concurrent association between autistic traits and receptive language abilities. Internet-based language tests were completed by ~3,000 pairs of twins, while autistic traits were assessed via parent ratings. Twin model fitting explored the association between these measures in the full sample, while DeFries-Fulker analysis tested these associations at the extremes of the sample. Phenotypic associations between language ability and autistic traits were modest and negative. The degree of genetic overlap was also negative, indicating that genetic influences on autistic traits lowered language scores in the full sample (mean genetic correlation = −0.13). Genetic overlap was also low at the extremes of the sample (mean genetic correlation = 0.14), indicating that genetic influences on quantitatively defined language difficulties were largely distinct from those on extreme autistic traits. Variation in language ability and autistic traits were also associated with largely different nonshared environmental influences. Language and autistic traits are influenced by largely distinct etiological factors. This has implications for molecular genetic studies of ASC and understanding the etiology of ASC. Additionally, these findings lend support to forthcoming DSM-5 changes to ASC diagnostic criteria that will see language difficulties separated from the core ASC communication symptoms, and instead listed as a clinical specifier.

## INTRODUCTION

Autism Spectrum Conditions (ASC) are neurodevelopmental conditions characterized by atypical social and communication abilities, and by repetitive, restricted patterns of behavior and interests [American Psychiatric Association [APA], 2013]. Twin studies suggest that both clinically assessed ASC and subclinical traits characteristic of ASC are highly heritable [Ronald & Hoekstra, 2011], yet the specific molecular genetic basis of ASC has proven challenging to elucidate. For instance, it has been suggested that multiple genes underlie ASC, and that potentially different genetic causes may be associated with each individual case [Abrahams & Geschwind, 2008; Geschwind, 2011].

Since the pioneering work of Bartak et al. (1975), a plethora of research has examined language abilities in individuals with ASC, suggesting some differences in pragmatic [e.g. Taylor et al., 2013], figurative language [e.g. Landa and Goldberg, 2005], syntactic [e.-g. Eigsti et al., 2007], and vocabulary [e.g. Norbury, 2005] ability across individuals with and without ASC. The emphasishis to historically placed on language impairments in ASC is further exemplified in the DSM-IV (APA, 1994) criteria for ASC, whereby the presence of language impairments was the core criteria for distinguishing autistic disorder from Asperger Syndrome. Given this, many molecular genetic studies have questioned whether ASC is associated with specific genetic variants associated with language impairment, for example CNTNAP2 [e.g. Alarcón et al., 2008; Arking et al., 2008], FOXP2 [e.g. Newbury et al., 2002], and SHANK3 [Durand et al., 2007].

While candidate gene studies yielded some initially promising findings, associations between ASC and variants associated with language impairment have yet to replicate in genome-wide association studies [e.g. Ronald et al., 2010; Connolly et al., 2013]. Furthermore, the role of language impairments in the ASC phenotype have been called into question by evidence to suggest that considerable variability exists in many language skills in individuals with ASC, particularly with regard to structural skills such as syntax [e.g. Whyte et al., 2013] and vocabulary [e.g. Whitehouse et al., 2007a]. Indeed, a single language profile of impairment seems insufficient to adequately characterize individuals with ASC [Kjelgaard & Tager-Flusberg, 2001]. It therefore seems important to establish whether or not ASC and language abilities do, in fact, have a shared aetiological basis.

The aim of the present study was hence to investigate the extent to which traits characteristic of ASC and language ability share genetic and environmental influences with one another using the classical twin design. One twin study to date suggested that autistic traits at age 8 and expressive language in early childhood share limited genetic and environmental influences with one another in the general population [Dworzynski et al., 2007] and at the extremes of the general population [Dworzynski et al., 2008]. Yet these studies did not employ concurrently collected data. Additionally, it is unknown whether the same findings would emerge between autistic traits and receptive language skills given that individuals with ASC often exhibit more difficulty with receptive than expressive language tasks [Hudry et al., 2010].

We hence explored the aetiological associations between autistic traits and four different receptive language skills in a general population twin sample. We also aimed to test whether similar associations would emerge across three core autistic trait domains (social and communication atypicalities, and repetitive, restricted behaviors and interests), and whether similar associations would be present at the extremes of the general population. While language impairments are not universal in ASC, a considerable proportion of individuals with ASC do appear to present with language difficulties, particularly in pragmatic domains. We hence hypothesized that autistic traits and receptive language would share a considerable degree of their aetiological influences with one another in the full sample and at the extremes. We also hypothesized that communication atypicalities characteristic of ASC would display the strongest degree of aetiological overlap with receptive language.

## METHOD

### Participants

The Twins Early Development Study (TEDS) is a population-representative, longitudinal, community sample of twins born in England and Wales between 1994–1996 [Haworth et al., 2013]. Parents of 12,666 12-year-old participants completed and returned questionnaires assessing traits of autism, and 8690–9310 individual twins completed four language tests. Participants were excluded if they displayed severe genetic conditions, including Fragile X syndrome and cystic fibrosis, or chromosomal abnormalities, including Down Syndrome and cerebral palsy. This resulted in the removal of 122 participants from the analyses. Participants were further excluded if first contact or zygosity data were missing, and if English was not the primary language spoken in the home, leaving 4764 twin pairs with autistic trait data, and 3222 pairs with data from at least one language measure. Participants with a confirmed ASC diagnosis (N = 71) were not excluded from the analyses. A total of 35 participants with ASC had language data available. Zygosity was assigned using DNA testing and parental observation of twin resemblance [Price et al., 2000]. Sample frequencies by zygosity are provided in Table II. Written informed consent was provided prior to participation.

### Measures

#### Traits of autism

Parents completed the Childhood Autism Spectrum Test (CAST [Scott et al., 2002]), comprising 30 ‘yes/no’ questions (the original version contains 31 questions; however, one age-inappropriate item was removed). The maximum possible score was 30; a score of 15 or above maximizes sensitivity (100%) and specificity (97%) to an ASC diagnosis [Williams et al., 2005]. In-line with prior studies [Ronald et al., 2006], the CAST was divided into three subscales corresponding to DSM-IV-TR [APA, 2000] autism symptoms: social; communication; and repetitive, restricted behaviors and interests (RRBI).

#### Receptive Language

Receptive language abilities were assessed using four internet-based, self-report measures. Internet-based testing offers the considerable advantage of allowing vast data to be collected. Validity of the in-person forms of these tests is described below; as such only indirect information on validity of the internet versions is available. Similar internet-based measures in mathematics and reading abilities administered to the TEDS sample at age 12 correlated with results obtained from the in-person versions of the tests [Haworth et al., 2007]. In all tests, audio streaming was used so that reading ability did not limit performance.

##### Figurative Language

The Figurative Language subtest of the Test of Language Competence [FL; Wiig et al., 1989] requires one to understand the non-literal meaning of a word alongside its literal meaning. Participants were read a sentence, and were then asked to select the correct interpretation of the sentence from a choice of four possibilities. The in-person version of the test correlates 0.62–0.78 with similar measures of language ability, and has 96% sensitivity in identifying language impaired individuals.

##### Pragmatics

The Making Inferences subtest of the Test of Language Competence [Pragmatics; Wiig et al., 1989] involves participants being read a description of an event; they are then asked to make a permissible inference about the cause of the event by answering a multiple-choice question about the causes of the event. The validity of the in-person form of this test is as above for FL.

##### Syntax

Participants completed the Listening Grammar subtest of the Test of Adolescent and Adult Language [Syntax; Hammill et al., 1994]. Participants were read three sentences, and were asked to select which two of the sentences had the same meaning. The in-person form of the measure displays correlations of 0.59–0.83 with similar measures of language. It also has 89% sensitivity for identifying individuals with language difficulties.

##### Vocabulary

Participants completed a multiple-choice adaptation of the vocabulary subtest of the Wechsler Intelligence Scales for Children [Vocabulary; Wechsler, 1992]. Participants were read a word, and then had to select the correct definition(s) of the word. The in-person test correlates 0.55–0.87 with similar measures of language; it is also discriminates effectively between individuals with low and high language ability.

### Data Analysis

#### Full Sample

Phenotypic associations in the full sample were explored using Pearson correlations between the measures (phenotypic correlations; r_ph_). One twin was randomly selected per pair when computing these correlations to account for the non-independence of twin data.

Twin analyses of the full sample aimed to estimate the degree of genetic and environmental overlap between continuous autistic traits and language abilities. Twin models estimate genetic influences on a phenotype, termed ‘heritability’, which can be divided into additive genetic influences (‘A’) and non-additive genetic influences (‘D’), arising from interacting alleles within loci. Environmental influences are also estimated, and include shared (‘C’) environmental influences, which are common to both twins in pair, heightening their similarity, and nonshared (‘E’) environmental influences, which differ between twins and create cross-twin dissimilarity.

Analyses began with cross-twin correlations, which indicate the extent of genetic and environmental influences, derived separately for MZ and DZ twins. MZ twins are assumed to share all of their segregating DNA code, while DZ twins are assumed to share ~50%. When MZ cross-twin correlations exceed DZ cross-twin correlations, A is indicated; E is indicated where the MZ correlation is less than unity. C is implicated if the DZ cross-twin correlation is at least half the MZ statistic. Where the DZ cross-twin correlation is less than half the MZ statistic, D is implicated.

Cross-trait cross-twin correlations, which correlate one twin’s score on one measure with their co-twin’s score on another, assessed etiological contributions to covariance between phenotypes. Cross-trait cross-twin correlations cannot exceed r_ph_ between traits. If the MZ cross-trait cross-twin correlation exceeds the DZ cross-trait cross-twin correlation, A influences on covariance are implied. Influences of E on covariance are indicated if the MZ correlation is less than the phenotypic correlation, while C is indicated when the DZ cross-trait cross-twin correlation is greater than half the MZ statistic. D is implicated when the DZ cross-trait cross-twin correlation is less than half the MZ statistic.

Structural equation twin model fitting was used to estimate A, C, D, and E. A Cholesky decomposition, presented here as a mathematically equivalent correlated factors solution [Loehlin, 1996], was fitted to data. C and D cannot be simultaneously estimated in this decomposition, hence only A, E, and C or D were estimated. Estimates of E include measurement error. A, C or D, and E are estimated for each phenotype, along with etiological correlations between phenotypes. A genetic correlation (r_g_) is calculated, and falls between −1 and 1. Wherer r_g_ = 1 or −1, all additive genetic influences are common to two phenotypes, while if r_g_ = 0, then all these influences are independent across phenotypes. Shared environmental (r_c_),non-additive genetic (r_d_), and nonshared environmental (r_e_) correlations were also computed, and operate in the same manner.

An additional statistic is bivariate heritability, which estimates the proportion of the phenotypic correlation explained by additive genetic influences, and is calculated:

$$(\sqrt{a_{1}}\times r_{g}\times \sqrt{a_{2}})÷\;r_{ph}$$

*a*_1_ and *a*_2_ are A for the first and second phenotype respectively, r_g_ is the genetic correlation between them, and r_ph_ is the phenotypic correlation. The extent of C, E, and D influences on the phenotypic correlation can be calculated in a similar manner.

Fits of Cholesky decompositions were compared with that of saturated models of the observed data using the likelihood-ratio test. The -2LL fit statistic was calculated for each model. The difference in -2LL between two models is χ^2^ distributed, with degrees of freedom (df) equivalent to the difference in number of parameters between two models, enabling a statistical comparison of fit. Significant χ^2^ results indicate that a given model is a poorer fit relative to the comparison model. Akaike’s Information Criterion (AIC), calculated χ^2^ - (2 × df), further assessed model fit. Lower values reflect better fitting models. Each model was fitted with estimates equated across sexes and quantitative sex limitation, which assumes the same etiological influences operate to differing extents in each sex.

The best fitting full model was selected using AIC. Within the best-fitting model, nested models were tested by dropping certain parameters by constraining them to equal zero. Nested models were tested with likelihood-ratio tests.

The CAST and its three subscales were log transformed for positive skew. Two language measures, Pragmatics and Vocabulary, were also skewed, and hence log transformed (see Table I). The mean effects of sex and age were regressed out of the scales in-line with standard behavioral genetic procedures [McGue & Bouchard, 1984]. Models were fitted to the language measures and full-scale CAST, and subsequently CAST subscales, using Mx [Neale et al., 2003]. Only same-sex pairs of twins were included in the analyses.

### Analysis of Extreme-Scoring Groups

Data from extreme-scoring groups within the sample were analysed to test the degree of genetic overlap between extreme autistic traits and language difficulties. All scales were z-transformed. Probands were defined on the basis of scoring within the highest 5% of the CAST distributions, or the lowest 5% of the language ability distributions. Subsequently, more extreme thresholds were employed; the highest 2.5% of the CAST distributions and lowest 2.5% of the language score distributions.

#### Phenotypic Associations

Phenotypic group correlations measure the relationship between two phenotypic scores in extreme-scoring groups. They were calculated by dividing the mean proband z-score on one measure with the mean proband z-score on the measure used to select probands. These estimates provide an indication of the extent of the phenotypic association between the measure used to select probands and proband quantitative scores on the second measure of interest.

#### Univariate DeFries-Fulker Extremes Analysis

DeFries-Fulker extremes analysis estimates heritability of extreme scores through regression-based analyses of means [DeFries & Fulker, 1985]. Scores on all measures were transformed so that the proband mean was 1, and the population mean was 0. Transformed co-twin means can be interpreted as twin group correlations, similar to cross-twin correlations; if the transformed DZ co-twin mean regresses toward the population mean more than the transformed MZ co-twin mean, genetic influences on extreme scores are indicated. Group heritability (h_2g_), the genetic contribution to extreme scores, was then estimated by fitting the following regression equation to the data:

$$C=\beta_{1}P+\beta_{2}R+A$$

*C* is co-twin scores on the measure of interest, *β*_1_*P* is the coefficient for proband scores on the same measure, *β*_2_*R* is the coefficient for zygosity, and *A* is the regression constant. *β*_2_*R* equals twice the difference between the transformed MZ co-twin mean and transformed DZ co-twin mean, and is an estimate of h_2g_. This should not exceed the transformed MZ co-twin mean, but may in the instance that non-additive genetic influences operate. Whenever this occurred, h_2g_ was constrained to equal transformed MZ co-twin mean.

#### Bivariate DeFries-Fulker Analysis

DeFries-Fulker analysis can be extended to examine genetic overlap between extreme scores on two measures [Light & DeFries, 1995]. Probands were selected on the basis of extreme scores on one measure, the selection variable. Genetic overlap with other phenotypes was explored by examining the relationship between the proband’s score on the selection variable and their co-twin’s score on another (the outcome variable). Transformed scores, as detailed above, were used in these analyses; genetic overlap is indicated when transformed DZ co-twin mean on the outcome variable more closely resembles the population mean of 0 than the transformed MZ co-twin mean.

Bivariate DeFries-Fulker analysis also estimates bivariate heritability (h_2.xy_), which indicates the degree of genetic influences on the selection variable that also influence the outcome variable. h_2.xy_ is bi-directional, in that it could, for example, be used to explore the relationship between the CAST and TOAL using the CAST as the selection variable and TOAL as the outcome, and vice-versa.

The bivariate DeFries-Fulker regression equation is as follows:

$$C_{y}=\beta_{1}P_{x}+\beta_{2}R+A$$

*C_y_* is co-twin scores on the outcome variable, *β*_1_*P_x_* is the partial regression on proband scores on the selection variable, *β*_2_*R* is the partial regression on zygosity, and *A* is the regression constant. *β*_2_*R* estimates h_2.xy_, which is capped at the transformed MZ co-twin mean on the outcome variable. The ratio of h_2.xy_ to the phenotypic group correlation between phenotypes indicates the proportion of the correlation explained by additive genetic factors. Where h_2.xy_ exceeds the phenotypic group correlations, this can indicate non-additive genetic influences [Dworzynski et al., 2008].

Calculating h_2.xy_ in both directions allows a genetic correlation (r_g_) to be calculated, which estimates genetic overlap between extreme scores [Knopik et al., 1997]:

$$\sqrt{\frac{\left(\beta_{xy}\times \beta_{yx}\right)}{\left(\beta_{x}\times \beta_{y}\right)}}$$

where *β_xy_* is bivariate heritability using the first variable of interest as the selection variable, *β_yx_* is the reverse, and *β_x_* and *β_y_* are h_2g_ estimates for each phenotype. All regression equations included sex and age.

## RESULTS

See Table I for descriptive statistics.

### Full Sample

Phenotypic correlations between the CAST and language measures were modest, and were −0.14 (Syntax), −0.15 (FL, Pragmatics), and −0.16 (Vocabulary) (*P* < 0.01). The mean phenotypic correlations between the CAST subscales and language measures were −0.08 (social), −0.06 (RRBI), and −0.18 (communication; see online appendix).

Twin correlations are presented in Table II. MZ cross-twin correlations all exceeded DZ cross-twin correlations, suggesting additive genetic influences (A) on all phenotypes. Non-additive genetic (D) and nonshared environmental influences (E) were suggested for the CAST and its subscales. For all language measures, shared environmental (C) and E were indicated. Cross-trait cross-twin correlations were all modest. For the most part, MZ correlations did not exceed DZ correlations, implying minimal influence of A on the covariance. MZ cross-trait cross-twin correlations were lower than the phenotypic correlations (r_ph_), suggesting E influences. Modest C influences were also implicated.

An ACE model with quantitative sex limitation best fit the full-scale CAST and language data, χ^2^_160_ = 216.02, p < 0.01, AIC = −103.98; all fit statistics are provided in Table III. Parameter estimates are given in Table IV.

Genetic correlations (r_g_) ranged from 0.01 to −0.18, suggesting few common A influences on language and autistic traits. Non-shared environmental correlations (r_e_) was also low, ranging from −0.08 to −0.01. Shared environmental overlap (r_c_), however, was higher (−0.43–−0.99), although confidence intervals were wide (see Table III).

While there was limited covariance between autistic traits and language, the bivariate heritability and environment estimates suggested that, in both sexes, the majority of r_ph_ between these measures was explained by shared environmental influences. The only exception was r_ph_ between autistic traits and FL in males, which was largely explained by additive genetic influences.

An AE model with quantitative sex limitation best fit the CAST subscales and language measures. All CAST subscales displayed high A influences (0.68–0.72), while was E modest (0.28–0.32). Etiological overlap between the social subscale and language was low, r_g_ =−0.13–0.01; r_e_ = −0.05–0.05. For RRBIs, r_g_ = −0.14–0.01 and r_e_ = −0.14–0.01. These estimates were slightly higher for autistic communication traits: r_g_ = −0.18–−0.05, r_e_ = −0.06–0.03.

### Extreme-Scoring Groups

Within the highest 5% of the CAST distribution, phenotypic group correlations with the language measures were similar to the full sample: −0.13 (FL), −0.14 (Syntax), and −0.15 (Pragmatics and Vocabulary). In the highest 5% of the CAST subscale distributions, these ranged from −0.07–−0.17. Within the highest 2.5% of the CAST distribution, phenotypic correlations were −0.11 (FL and Vocabulary), −0.12 (Pragmatics), and −0.13 (Syntax). Within the highest 2.5% of the CAST subscales, these values fell between −0.06–−0.17.

#### Univariate Analyses

Transformed means are presented in the supplementary materials. Across all measures in both extreme-scoring groups, the transformed DZ co-twin means regressed towards the population mean to a greater extent than the transformed MZ co-twin means, suggesting genetic influences on extreme scores. Group heritability (h_2g_) estimates were substantial for the CAST and its subscales, ranging from 0.69 to 0.76, and modest for the language measures, ranging from 0.18 to 0.55.

#### Bivariate Analyses

Bivariate heritability (h_2.xy_) suggested the proportion of the modest phenotypic group correlations that could be explained by additive genetic influences ranged from none to the entire phenotypic group correlation. There was evidence of non-additive genetic influences on some phenotypic group correlations. Across all CAST subscales and language measures, r_g_ was modest. In the 5% extreme-scoring group, the highest estimate was 0.32 (CAST Communication – Vocabulary), while the lowest was 0.01 (CAST Social – FL). In the 2.5% analysis, r_g_ fell between 0 (full-scale CAST and Vocabulary) and 0.26 (CAST Communication – Vocabulary). The results of the DeFries-Fulker analyses are fully presented in the online appendices.

## DISCUSSION

We aimed to examine the concurrent association between autistic traits and receptive language skills in a general population sample. The historically advocated link between ASC and language led us to expect relatively strong phenotypic and etiological associations. Contrary to this, all four language measures displayed weak etiological and phenotypic links with autistic traits, which extended across three autistic trait domains and to the extremes of the general population. This pattern extended to communication atypicalities characteristic of ASC, which were expected to show stronger overlap with language.

As mentioned previously, the historic link between ASC and language has motivated some molecular genetic studies to question the role of variants thought to associated with language in ASC [e.g. Alarcón et al., 2008]. In our study, it is noteworthy that more covariance between autistic traits and language could actually be explained by shared environmental influences, as indicated by the bivariate heritability and environment estimates. Additive genetic overlap was also very low, suggesting different genetic influences on language and autistic traits. This could partially explain why linkage [e.g. Spence et al., 2006], case-control association [e.g. Toma et al., 2012], and genome-wide association studies [e.g. Connolly et al., 2013] have not consistently replicated associations between variants linked with language impairment and ASC.

There are two further possibilities regarding these findings. First, they could be taken as adding evidence to the fractionable autism triad hypothesis [Happé et al., 2006; Happé & Ronald, 2008]. This hypothesis posits that the core ASC symptom domains, social and communication atypicalities and repetitive, restricted behaviors and interests, have different causes to one another. However, as Bishop (2010) points out, within the communication symptom domain it is worth drawing a distinction between pragmatic aspects of language, covered by the CAST Communication subscale, and more structural components of language. Hence, if one is to assume that language difficulties form a core component of the ASC phenotype, then these findings support the notion that they arise via different causes to the rest of the core ASC symptoms.

Alternatively, these findings could be taken as quantitative genetic support for the separation of language difficulties from the ASC phenotype. In the DSM-5 [APA, 2013], language difficulties have been removed from the core ASC symptoms, and instead have been listed as clinical specifiers. Our findings support this adaptation; language, both ability in the full sample and quantitatively defined disability at the extremes, showed weak phenotypic and etiological associations with autistic traits. This suggests that language and autistic traits can be separated. Indeed, this notion is further supported by evidence from family studies [e.g. Lindgren et al., 2009] and studies of singletons [e.g. Whitehouse et al., 2007b], including those that suggest that no single profile of language ability is adequate to characterise individuals with ASC [Kjelgaard & Tager-Flusberg, 2001].

A notable exception to these findings was shared environmental overlap, which was substantially higher than additive genetic and nonshared environmental overlap. However, it is important to note the wide confidence intervals around the shared environmental correlations (see Table III), which often overlapped with zero. Additionally, shared environmental influences account for a small proportion of variance in each measure, meaning that shared environmental influences that are common to autistic traits and language only account for a small proportion of variance in each individual phenotype.

As with any study, our research was not without limitations. Autistic traits were assessed by a single rater; future research should test whether these findings extend to self- and teacher-reported autistic traits. Some researchers question the extent to which findings from twins generalize to singletons. However, recent studies suggest that twinning does not elevate autistic trait scores [Curran et al., 2011]. Additionally, the early language delay some-times seen in twins disappears by middle childhood [Dale et al., 2010].

It is a limitation that the internet-based versions of the language tests used here have yet to be extensively validated. While Haworth et al. (2007) reported that internet and in-person versions of mathematics and reading ability correlate with one another, no such information is available for these language measures, and future work should test the validity of these measures. There are, however, some reasons to feel reassured that the weak associations seen in this study were not simply due to the validity of the language measures. Firstly, Dworzynski et al. (2007, 2008) reported that aetiological and phenotypic overlap between autistic traits and validated, in-person expressive vocabulary tests was still low (albeit, with the two measures administered at different ages). Additionally, evidence from family studies that have used validated language assessments also hints at very little aetiological overlap between ASC and language abilities [e.g. Lindgren et al., 2009; Kalnak et al., 2012].

Our large sample meant that administering in-depth clinical assessments was not feasible. This was not, however, necessarily a limitation; trait-based questionnaires can complement research with clinically based samples by enabling the large samples required to perform twin modelling to be studied, whilst avoiding biases associated with clinical samples. There is also evidence of continuity between heritability of autistic traits in the general population and in extreme-scoring groups, including those scoring at a comparable level with diagnosed samples [Robinson et al., 2011; Lundström et al., 2012].

In addition, our findings do not necessarily apply to ‘syndromic’ ASC. Syndromic cases of ASC are associated with a known genetic cause [Abrahams & Geschwind, 2008], and often feature language impairments [Moss & Howlin, 2009]. It is possible that language difficulties in these cases are related to the known genetic cause. Hence, our findings most likely apply to non-syndromic cases of ASC.

In conclusion, general population variation in autistic traits and receptive language ability are caused by largely different etiological factors. Additionally, quantitatively defined language difficulties appear to be caused by different additive genetic influences to extreme scores on autistic trait measures. This suggests that molecular genetic studies of ASC and language impairments will produce largely discrepant findings. Furthermore, these findings lend support to the imminent removal of language impairments from the core ASC symptoms, instead being listed as a clinical specifier.

## Supplementary Material

Supplementary Tables 1-6

## Figures and Tables

**TABLE I:** Descriptive Statistics for the CAST and Language Measures

Measure	Number of items	Maximum possible score	Cronbach’s α	Skew^i^	*x* Full sample (SD)	*x* 5% Extreme group (SD)	*x* 2.5% Extreme group (SD)
CAST^a^	30	30	0.73	1.57 (−0.43)	4.79 (3.47)	14.08 (3.29)	16.06 (3.11)
CAST Social^b^	11	11	0.54	1.42 (−0.01)	1.55 (1.47)	4.37 (2.13)	5.07 (2.25)
CAST RRBI^c^	7	7	0.49	0.96 (−0.05)	1.36 (1.47)	3.47 (1.51)	3.85 (1.55)
CAST Communication^d^	12	12	0.64	1.36 (0.02)	1.88 (1.87)	6.24 (2.00)	7.14 (2.00)
Figurative Language^e^	11	11	0.67	−0.20	6.13 (2.54)	5.23 (2.75)	5.21 (2.65)
Pragmatics^f^	11	33	0.71	−0.77 (−0.56)	25.17 (4.62)	23.28 (5.30)	23.34 (5.12)
Syntax^g^	35	35	0.94	0.21	16.22 (9.30)	12.74 (8.93)	12.42 (8.63)
Vocabulary^h^	60	60	0.88	−0.96 (−0.34)	39.21 (10.49)	34.98 (10.49)	35.32 (11.97)

a CAST: Childhood Autism Spectrum Test.

b CAST Social: Social atypicalities subscale of the CAST.

c CAST Communication: Communication difficulties subscale ofthe CAST.

d CAST RRBI: Repetitive, restricted behaviours and interests subscale of the CAST.

e Figurative Language subtest of the Test of Language Competence.

f Making Inferences subtest of the Test of Language Competence.

g Listening Grammar subtest of the Test of Adolescent and Adult Language.

h Vocabulary subtest of the Wechsler Intelligence Scales for Children.

i Skew statistics are first given for the untransformed scale; values given in brackets are for the log transformed scale where such transformations were performed.

**TABLE II:** Cross-Twin and Cross-Trait Cross Twin Correlations

		CAST^b^		Figurative Language^c^		Pragmatics^d^		Syntax^e^		Vocabulary^f^	
Zygosity	N Pairs	ICC^g^	95% CI^h^	ICC^g^	95% CI^h^	ICC^g^	95% CI^h^	ICC^g^	95% CI^h^	ICC^g^	95% CI^h^
Univariate Cross-Twin Correlations											
MZM^a^	1113	0.78	0.76/0.79	0.53	0.49/0.57	0.42	0.37/0.47	0.41	0.36/0.45	0.44	0.40/0.69
DZM^a^	1102	0.27	0.22/0.31	0.36	0.31/0.41	0.30	0.25/0.35	0.31	0.25/0.36	0.32	0.27/0.37
MZF^a^	1293	0.74	0.72/0.76	0.55	0.52/0.58	0.44	0.40/0.48	0.48	0.44/0.51	0.42	0.38/0.46
DZF^a^	1188	0.42	0.38/0.46	0.37	0.33/0.41	0.30	0.26/0.35	0.32	0.27/0.37	0.29	0.24/0.33

	CAST–Figurative Language		CAST–Pragmatics		CAST–Syntax		CAST–Vocabulary	
Zygosity	ICC^g^	95% CI^h^	ICC^g^	95% CI^h^	ICC^g^	95% CI^h^	ICC^g^	95% CI^h^
Bivariate Cros-Trait Cros-Twin Correlations								
MZM^a^	−0.13	−0.19/−0.08	−0.10	−0.15/−0.04	−0.06	−0.12/0.00	−0.09	−0.15/−0.04
DZM^a^	−0.11	−0.17/−0.06	−0.15	−0.20/−0.09	−0.08	−0.14/−0.02	−0.05	−0.11/−0.01
MZF^a^	−0.13	−0.18/−0.09	−0.11	−0.16/−0.06	−0.09	−0.13/−0.04	−0.09	−0.14/−0.04
DZF^a^	−0.16	−0.21/−0.11	−0.14	−0.20/−0.09	−0.09	−0.14/−0.03	−0.06	−0.11/−0.01

a MZM: monozygotic males; DZM: dizygotic males; MZF: monozygotic females; DZF: dizygotic females.

b CAST: Childhood Autism Spectrum Test.

c Figurative Language subtest of the Test of Language Competence.

d Making Inferences subtest of the Test of Language Competence.

e Listening Grammar subtest of the Test of Adolescent and Adult Language.

f Vocabulary subtest ofthe Wechsler Intelligence Scales for Children.

g ICC: intraclass correlation coefficient.

h 95% CI: 95% confidence intervals.

**TABLE III:** Twin Model Fit Statistics

				Comparison with Saturated Model				Comparison with Best Fitting Full Model			
Model	2LL^a^	df^b^	Parameters	Δ * χ * ^2c^	Δdf^d^	*p* ^e^	AIC^f^	Δ * χ * ^2^	Δdf	*p*	AIC
**Sexes Equated**											
Saturated^g^	76817.71	29654	130	—	—	—	—	—	—	—	—
ACE^h^	76944.08	29725	50	126.37	80	<0.001	−33.63	—	—	—	—
ADE^i^	76995.10	29725	50	177.39	80	<0.001	17.39	—	—	—	—
**Quantitative Sex Limitation**											
Saturated	76672.36	29515	260	—	—	—	—	—	—	—	—
ACE*	76888.36	29675	100	216.02	160	<0.01	−103.98	—	—	—	—
ADE	76950.33	29675	100	277.97	160	<0.001	−42.03	—	—	—	—
AE^J^	76962.25	29705	70	289.89	190	<0.001	−70.11	77.86	30	<0.001	13.86
CE^J^	77423.37	29705	70	751.01	190	<0.001	391.01	534.98	30	<0.001	474.98
E^J^	80402.56	29735	40	3730.20	220	<0.001	3290.20	3514.17	60	<0.001	3394.17

* Model chosen as best-fitting based on lowest AIC value.

a Fit statistics, which is −2 times the log-likelihood of the data.

b df: degrees of freedom.

c Change in −2LL between two models, which is distributed χ^2^.

d Change in df between two models, which is equal to the difference in the number of parameters between two models.

e *p*-value derived from the likelihood-ratio test, which is based on both the change in −2LL and df between two models.

f AIC: Akaike’s Information Criteria, an alternative fit statistic. Lower, preferably negative, values reflect better fitting models.

g Saturated model of the observed means, variance, and covariance in the data.

h A: additive genetic influence; C: shared environmental influence; E: residual term, incorporating nonshared environmental influences and measurement error.

i D: non-additive genetic influence.

J AE, CE, and E models are nested within the ACE model and test the significance of A and C parameters within the model.

**TABLE IV:** Parameter Estimates From the ACE Correlated Factors Solution With Quantitative Sex Limitation

	A		C		E	
	Male	Female	Male	Female	Male	Female
Variance Components Estimates^a^						
CAST^d^	0.73 (0.66/0.78)	0.52 (0.49/0.61)	0.04 (0.01/0.11)	0.25 (0.16/0.30)	0.22 (0.20/0.25)	0.23 (0.21/0.25)
Figurative Language^e^	0.39 (0.24/0.53)	0.39 (0.34/0.49)	0.12 (0.01/0.25)	0.12 (0.44/0.53)	0.49 (0.44/0.54)	0.48 (0.44/0.53)
Pragmatics^f^	0.18 (0.07/0.31)	0.25 (0.15/0.35)	0.15 (0.04/0.25)	0.12 (0.10/0.23)	0.67 (0.61/0.73)	0.63 (0.58/0.68)
Syntax^g^	0.25 (0.11/0.39)	0.22 (0.11/0.36)	0.18 (0.07/0.25)	0.21 (0.08/0.32)	0.57 (0.51/0.63)	0.57 (0.53/0.62)
Vocabulary^h^	0.27 (0.13/0.42)	0.33 (0.19/0.44)	0.19 (0.07/0.31)	0.12 (0.07/0.19)	0.54 (0.48/0.60)	0.55 (0.50/0.60)

	r_A_		r_C_		r_E_	
	Male	Female	Male	Female	Male	Female
Aetiological						
Correlations^b^						
CAST-Figurative Language	−0.18 (−0.32/−0.04)	−0.10 (−0.09/0.01)	−0.71 (−0.99/−0.05)	−0.83 (−1.00/−0.39)	−0.08 (−0.16/−0.01)	−0.02 (−0.03/0.08)
CAST-Pragmatics	−0.12 (− 0.33/−0.11)	−0.15 (−0.29/−0.09)	−0.99 (−1.00/0.73)	−0.94 (−1.00/−0.44)	0.01 (−0.07/0.08)	−0.01 (−0.05/0.02)
CAST-Syntax	−0.10 (−0.26/0.11)	−0.16 (−0.44/−0.11)	−0.77 (−0.99/−0.05)	−0.43 (−0.86/−0.10)	−0.02 (−0.10/0.05)	−0.04 (−0.11/0.04)
CAST-Vocabulary	−0.18 (−0.38/−0.04)	−0.13 (−0.36/−0.07)	−0.67 (−1.00/0.03)	−0.59 (−0.81/−0.21)	−0.04 (−0.11/0.04)	−0.03 (−0.06/0.05)

	Bivariate A		Bivariate C		Bivariate E	
	Male	Female	Male	Female	Male	Female
Bivariate Heritability and Environment^c^						
CAST-Figurative Language	0.56	0.25	0.28	0.70	0.17	0.05
CAST-Pragmatics	0.33	0.24	0.67	0.76	0.00	0.00
CAST-Syntax	0.33	0.31	0.58	0.63	0.08	0.06
CAST-Vocabulary	0.53	0.31	0.40	0.63	0.07	0.06

a These estimates divide the phenotypic variance into additive genetic (A), shared environmental (C) and nonshared environmental (E) components. Statistics are expressed as a proportion of the phenotypic variance explained by A, C, and E.

b The degree of aetiological overlap between two phenotypes, including additive genetic (r_A_), shared environmental (r_C_), and nonshared environmental (r_E_) correlations.

c These estimates divide the phenotypic covariance between two phenotypes into A, C, and E, and are expressed as the proportions ofthe phenotypic correlations given in the text explained by A, C, and E.

d CAST: Childhood Autism Spectrum Test.

e Figurative Language subtest of the Test of Language Competence.

f Making Inferences subtest of the Test of Language Competence.

g Listening Grammar subtest of the Test of Adolescent and Adult Language.

h Vocabulary subtest of the Wechsler Intelligence Scales for Children.
