# Supplementary material for: Language and Traits of Autism Spectrum Conditions: Evidence of Limited Phenotypic and Etiological Overlap
Source: Am J Med Genet B Neuropsychiatr Genet. Author manuscript; Available in PMC 2015 May 5. (PMC4419741; doi:10.1002/ajmg.b.32262)
Supplement: Supplementary Tables 1-6 [file NIHMS63232-supplement-Supplementary_Tables_1-6.docx]

**Taylor et al. AJMGB Electronic Appendices**

*Table 1 Phenotypic correlations for the full sample and phenotypic group correlations for extreme-scoring groups*

|  |  | Figurative Language | Making Inferences | TOAL ^a^ | Vocabulary ^b^ |
| --- | --- | --- | --- | --- | --- |
| CAST ^c^ | Full Sample ^g^ | -0.15** | -0.15** | -0.14** | -0.16** |
|  | Highest 5% ^h^ | -0.13 | -0.15 | -0.14 | -0.15 |
|  | Highest 2.5% ^i^ | -0.11 | -0.12 | -0.13 | -0.11 |
| CAST Social ^d^ | Full Sample | -0.07** | -0.08** | -0.08** | -0.09** |
|  | Highest 5% | -0.09 | -0.07 | -0.07 | -0.11 |
|  | Highest 2.5% | -0.06 | -0.06 | -0.09 | -0.10 |
| CAST RRBI ^e^ | Full Sample | -0.04** | -0.07** | -0.06** | -0.06** |
|  | Highest 5% | -0.08 | -0.10 | -0.09 | -0.09 |
|  | Highest 2.5% | -0.08 | -0.09 | -0.09 | -0.08 |
| CAST Communication^f^ | Full Sample | -0.20** | -0.17** | -0.16** | -0.18** |
|  | Highest 5% | -0.14 | -0.17 | -0.13 | -0.15 |
|  | Highest 2.5% | -0.16 | -0.15 | -0.15 | -0.17 |

*^a^ TOAL: Listening Grammar subtest of the Test of Adolescent and Adult Language*

*^b^ Vocabulary: Vocabulary subtest of the Wechsler Intelligence Scales for Children*

*^c^ CAST: Childhood Autism Spectrum Test*

*^d^ CAST Social: Social atypicalities subscale of the CAST*

*^e^ CAST RRBI: Repetitive, restricted behaviors and interests subscale of the CAST*

*^f^ CAST Communication: Communication atypicalities subscale of the CAST*

*^g^ Pearson correlations of the entire sample (** p<0.01)*

*^h^ Phenotypic group correlations for the highest 5% of CAST scale scorers. These are calculated by dividing the mean proband z-score on the selection measure by the mean proband z-score on the outcome measure. Note that these are directional; it would be possible to calculate them in the reverse direction, using language measures as selection variables and CAST scales as outcome variables.*

*^i^ Phenotypic group correlations, as described above, for the highest 2.5% of CAST scorers*

*Table 2 Fit statistics for multivariate twin models of the CAST subscales*

|  | | | | Comparison with Saturated Model ^a^ | | | | Comparison with Full ACE/ADE Model ^b^ | | | |
| --- | --- | --- | --- | --- | --- | --- | --- | --- | --- | --- | --- |
| Model | -2LL^c^ | df^d^ | Parameters | Δx^2 e^ | Δdf ^f^ | p ^g^ | AIC ^h^ | Δx^2^ | Δdf | p | AIC |
| Models with Sexes Equated ^i^ | | | | | | | | | | | |
| Saturated | 100550.62 | 37164 | 238 | ----- | ----- | ----- | ----- | ----- | ----- | ----- | ----- |
| ACE | 100769.77 | 37311 | 91 | 219.15 | 147 | <0.001 | -74.85 | ----- | ----- | ----- | ----- |
| AE | 100799.54 | 37339 | 63 | 248.92 | 175 | <0.001 | -101.08 | 29.77 | 28 | 0.38 | -26.23 |
| CE | 101492.82 | 37339 | 63 | 942.20 | 175 | <0.001 | 592.20 | 723.06 | 28 | <0.001 | 667.06 |
| E | 104445.70 | 37367 | 35 | 3895.08 | 203 | <0.001 | 3289.08 | 3675.93 | 56 | <0.001 | 3563.93 |
| ADE | 100769.68 | 37311 | 91 | 219.06 | 147 | <0.001 | -74.94 | ----- | ----- | ----- | ----- |
| Models with Quantitative Sex Limitation ^j^ | | | | | | | | | | | |
| Saturated | 100301.42 | 36926 | 476 | ----- | ----- | ----- | ----- | ----- | ----- | ----- | ----- |
| ACE | 100696.23 | 37220 | 182 | 394.81 | 294 | <0.001 | -193.19 | ----- | ----- | ----- | ----- |
| AE | 100748.75 | 37276 | 126 | 447.33 | 350 | <0.001 | -252.67 | 52.52 | 56 | 0.61 | -59.48 |
| CE | 101413.43 | 37276 | 126 | 1112.01 | 350 | <0.001 | 412.01 | 717.20 | 56 | <0.001 | 605.20 |
| E | 104406.99 | 37332 | 70 | 4105.57 | 406 | <0.001 | 3293.57 | 3710.76 | 112 | <0.001 | 3486.76 |
| ADE | 100697.98 | 37220 | 182 | 396.56 | 294 | <0.001 | -191.44 | ----- | ----- | ----- | ----- |

*^a^ Saturated models are an index of the observed data, modelling the means, variance, and covariance present in the observed data*

*^b^ A: Additive genetic influences; C: shared environmental influences; D: non-additive genetic influences; E: nonshared environmental influences*

*^c^ -2LL: fit statistic, two times the log-likelihood of the data*

*^d^ df: degrees of freedom*

*^e^ Δx^2^: change in -2LL between models, which is x^2^ distributed*

*^f^ Δdf: change in degrees of freedom between models, which is equivalent to the difference in number of parameters*

*^g^ p: p-value; p-values of less than 0.05 indicate a significant deterioration of fit between two models*

*^h^ AIC: Akaike’s Information Criterion, a fit statistic calculated Δx^2^ – (2 X Δdf)*

*^i^ Models with sexes equated provide a single estimate of each parameter*

*^j^ Models with quantitative sex limitation assume that the same etiological influences operate to differing extents in both sexes, and provide separate parameter estimates for males and females*

*Table 3 Parameter estimates for best-fitting multivariate model of the CAST subscales (95% confidence intervals in parentheses)*

| Variance components estimates | | | | |
| --- | --- | --- | --- | --- |
|  | A ^a^ | | E ^b^ | |
| Measure | Male | Female | Male | Female |
| CAST Social ^c^ | 0.72 (0.65-0.78) | 0.71 (0.66-0.73) | 0.28 (0.26-0.30) | 0.29 (0.21-0.33) |
| CAST RRBI ^d^ | 0.68 (0.61-0.69) | 0.69 (0.66-0.71) | 0.32 (0.30-0.34) | 0.31 (0.25-0.34) |
| CAST Communication ^e^ | 0.72 (0.67-0.78) | 0.71 (0.67-0.72) | 0.28 (0.24-0.30) | 0.29 (0.26-0.33) |
| Figurative Language ^f^ | 0.30 (0.14-0.35) | 0.26 (0.20-0.32) | 0.70 (0.59-0.77) | 0.74 (0.70-0.78) |
| Making Inferences ^g^ | 0.16 (0.10-0.20) | 0.14 (0.11-0.19) | 0.84 (0.80-0.90) | 0.86 (0.84-0.87) |
| TOAL ^h^ | 0.29 (0.21-0.33) | 0.26 (0.21-0.29) | 0.71 (0.66-0.73) | 0.74 (0.71-0.77) |
| Vocabulary ^i^ | 0.28 (0.24-0.33) | 0.24 (0.19-0.29) | 0.72 (0.69-0.75) | 0.76 (0.70-0.79) |
| Aetiological correlations | | | | |
|  | Additive Genetic Correlations | | Nonshared Environmental Correlations | |
| Measures | Male | Female | Male | Female |
| CAST Social – FL ^f^ | -0.06 (-0.11/0.02) | -0.04 (-0.09/-0.01) | -0.05 (-0.18/0.01) | -0.01 (-0.09/0.04) |
| CAST Social – MI ^g^ | -0.05 (-0.09/0.03) | 0.01 (-0.09/0.05) | 0.00 (-0.23/0.17) | -0.01 (-0.10/0.02) |
| CAST Social – TOAL ^h^ | -0.06 (-0.10/0.04) | -0.08 (-0.18/0.01) | -0.02 (-0.10/0.03) | 0.05 (-0.03/0.10) |
| CAST Social – Vocabulary ^i^ | -0.13 (-0.20/-0.01) | -0.07 (-0.15/-0.03) | -0.01 (-0.12/0.04) | -0.03 (-0.09/0.13) |
| CAST RRBI – FL | 0.01 (-0.10/0.13) | -0.01 (-0.13/0.03) | -0.08 (-0.12/-0/03) | 0.00 (-0.19/0.13) |
| CAST RRBI – MI | -0.14 (-0.30/-0.07) | -0.17 (-0.32/-0.11) | 0.07 (-0.02/0.14) | 0.04 (-0.08/0.09) |
| CAST RRBI – TOAL | -0.02 (-0.10/0.09) | -0.14 (-0.26/-0.09) | 0.00 (-0.33/0.17) | -0.02 (-0.16/0.04) |
| CAST RRBI – Vocabulary | -0.09 (-0.21/-0.02) | -0.07 (-0.12/-0.03) | 0.04 (-0.12/0.09) | -0.02 (-0.20/0.13) |
| CAST Comm. – FL | -0.18 (-0.30/-0.10) | -0.10 (-0.17/-0.05) | -0.05 (-0.17/-0.01) | -0.05 (-0.10/0.01) |
| CAST Comm. – MI | -0.14 (-0.28/-0.09) | -0.06 (-0.11/0.01) | -0.01 (-.21/0.04) | -0.06 (-0.12/-0.01) |
| CAST Comm. – TOAL | -0.14 (-0.27/-0.05) | -0.13 (-0.14/0.03) | 0.03 (-0.19/0.06) | -0.02 (-0.13/0.12) |
| CAST Comm. – Vocabulary | -0.11 (-0.21/-0.01) | -0.05 (-0.18/0.03) | 0.01 (-0.13/0.19) | 0.03 (-0.03/0.04) |

*^a^ Additive genetic influences*

*^b^ Nonshared environmental influences*

*^c^ Social atypicalities subscale of the CAST*

*^d^ Repetitive, restricted behaviors and interests subscale of the CAST*

*^e^ Communication atypicalities subscale of the CAST*

*^f^ Figurative Language subtest of the Test of Language Competence*

*^g^ Making Inferences subtest of the Test of Language Competence*

*^h^ Listening Grammar subtest of the Test of Adolescent and Adult Language*

*^i^ Vocabulary subtest of the Wechsler Intelligence Scales for Children*

***DeFries-Fulker Analyses***

*Table 4 Transformed means used in the DeFries-Fulker analysis*

| Univariate analysis | | | | | | | | |
| --- | --- | --- | --- | --- | --- | --- | --- | --- |
|  | 5% Extreme Groups ^a^ | | | | 2.5% Extreme Groups ^b^ | | | |
|  | Monozygotic Twins | | Dizygotic Twins | | Monozygotic Twins | | Dizygotic Twins | |
|  | Proband | Co-Twin | Proband | Co-Twin | Proband | Co-Twin | Proband | Co-Twin |
| CAST ^c^ | 1.00 | 0.76 | 1.00 | 0.31 | 1.00 | 0.73 | 1.00 | 0.23 |
| CAST Social ^d^ | 1.00 | 0.69 | 1.00 | 0.32 | 1.00 | 0.66 | 1.00 | 0.22 |
| CAST RRBI ^e^ | 1.00 | 0.72 | 1.00 | 0.33 | 1.00 | 0.71 | 1.00 | 0.32 |
| CAST Comm. ^f^ | 1.00 | 0.76 | 1.00 | 0.32 | 1.00 | 0.74 | 1.00 | 0.26 |
| FL ^g^ | 1.00 | 0.54 | 1.00 | 0.45 | 1.00 | 0.55 | 1.00 | 0.35 |
| MI ^h^ | 1.00 | 0.49 | 1.00 | 0.41 | 1.00 | 0.45 | 1.00 | 0.38 |
| TOAL ^i^ | 1.00 | 0.57 | 1.00 | 0.43 | 1.00 | 0.53 | 1.00 | 0.39 |
| Vocabulary ^j^ | 1.00 | 0.38 | 1.00 | 0.29 | 1.00 | 0.35 | 1.00 | 0.26 |
| Bivariate analysis | | | | | | | | |
|  | 5% Extreme Groups | | | | 2.5% Extreme Groups | | | |
|  | Monozygotic Twins | | Dizygotic Twins | | Monozygotic Twins | | Dizygotic Twins | |
| Selection Outcome | Proband | Co-Twin | Proband | Co-Twin | Proband | Co-Twin | Proband | Co-Twin |
| CAST – FL | 0.24 | 0.22 | 0.29 | 0.23 | 0.16 | 0.11 | 0.24 | 0.15 |
| CAST – MI | 0.18 | 0.16 | 0.23 | 0.19 | 0.12 | 0.08 | 0.19 | 0.10 |
| CAST – TOAL | 0.27 | 0.28 | 0.25 | 0.20 | 0.28 | 0.22 | 0.23 | 0.11 |
| CAST – Vocabulary | 0.21 | 0.21 | 0.20 | 0.11 | 0.20 | 0.18 | 0.15 | 0.03 |
| Social – FL | 0.20 | 0.17 | 0.18 | 0.18 | 0.12 | 0.08 | 0.12 | 0.21 |
| Social – MI | 0.11 | 0.12 | 0.12 | 0.13 | 0.08 | 0.06 | 0.10 | 0.12 |
| Social – TOAL | 0.18 | 0.19 | 0.12 | 0.14 | 0.18 | 0.13 | 0.15 | 0.15 |
| Social – Vocabulary | 0.17 | 0.17 | 0.12 | 0.09 | 0.18 | 0.15 | 0.12 | 0.09 |
| RRBI – FL | 0.20 | 0.21 | 0.16 | 0.16 | 0.12 | 0.13 | 0.11 | 0.11 |
| RRBI – MI | 0.13 | 0.08 | 0.16 | 0.13 | 0.09 | 0.05 | 0.12 | 0.11 |
| RRBI – TOAL | 0.19 | 0.18 | 0.14 | 0.11 | 0.14 | 0.13 | 0.13 | 0.09 |
| RRBI – Vocabulary | 0.16 | 0.12 | 0.10 | 0.08 | 0.13 | 0.10 | 0.08 | 0.07 |
| Comm. – FL | 0.24 | 0.22 | 0.35 | 0.30 | 0.25 | 0.25 | 0.31 | 0.15 |
| Comm. – MI | 0.19 | 0.13 | 0.27 | 0.18 | 0.11 | 0.12 | 0.28 | 0.14 |
| Comm. – TOAL | 0.21 | 0.19 | 0.29 | 0.18 | 0.25 | 0.20 | 0.32 | 0.14 |
| Comm. – Vocabulary | 0.18 | 0.18 | 0.24 | 0.11 | 0.28 | 0.27 | 0.22 | 0.01 |
| FL – CAST | 0.13 | 0.11 | 0.19 | 0.13 | 0.10 | 0.08 | 0.14 | 0.12 |
| FL – Social | 0.15 | 0.13 | 0.17 | 0.15 | 0.07 | 0.04 | 0.09 | 0.07 |
| FL – RRBI | 0.07 | 0.06 | 0.08 | 0.10 | 0.06 | 0.04 | 0.12 | 0.12 |
| FL – Comm. | 0.15 | 0.13 | 0.21 | 0.11 | 0.13 | 0.13 | 0.14 | 0.12 |
| MI – CAST | 0.13 | 0.11 | 0.30 | 0.23 | 0.13 | 0.05 | 0.22 | 0.16 |
| MI – Social | 0.14 | 0.11 | 0.23 | 0.18 | 0.11 | 0.04 | 0.10 | 0.10 |
| MI – RRBI | 0.12 | 0.10 | 0.20 | 0.19 | 0.12 | 0.05 | 0.17 | 0.14 |
| MI – Comm. | 0.12 | 0.11 | 0.29 | 0.21 | 0.13 | 0.06 | 0.25 | 0.16 |
| TOAL – CAST | 0.10 | 0.08 | 0.23 | 0.15 | 0.07 | 0.05 | 0.18 | 0.11 |
| TOAL – Social | 0.09 | 0.10 | 0.18 | 0.18 | 0.04 | 0.05 | 0.11 | 0.11 |
| TOAL – RRBI | 0.12 | 0.07 | 0.11 | 0.08 | 0.12 | 0.07 | 0.11 | 0.08 |
| TOAL – Comm. | 0.09 | 0.08 | 0.25 | 0.14 | 0.06 | 0.05 | 0.20 | 0.10 |
| Vocab. – CAST | 0.18 | 0.13 | 0.21 | 0.13 | 0.14 | 0.09 | 0.20 | 0.10 |
| Vocab. – Social | 0.19 | 0.14 | 0.23 | 0.16 | 0.10 | 0.07 | 0.20 | 0.09 |
| Vocab. – RRBI | 0.13 | 0.07 | 0.08 | 0.09 | 0.13 | 0.09 | 0.09 | 0.10 |
| Vocab. – Comm. | 0.19 | 0.15 | 0.20 | 0.10 | 0.15 | 0.11 | 0.19 | 0.09 |

*^a^ 5% extreme groups contain participants scoring within the highest 5% of the CAST scale distributions and lowest 5% of the language score distributions.*

*^b^ 2.5% extreme groups contain participants scoring within the highest 2.5% of the CAST scale distributions and lowest 2.5% of the language score distributions.*

*^c^ CAST: Childhood Autism Spectrum Test*

*^d^ CAST Social: Social atypicalities subscale of the CAST*

*^e^ CAST RRBI: Repetitive, restricted behaviors and interests subscale of the CAST*

*^f^ CAST Comm.: Communication atypicalities subscale of the CAST*

*^g^ FL: Figurative Language subtest of the Test of Language Competence*

*^h^ MI: Making Inferences subtest of the Test of Language Competence*

*^i^ TOAL: Listening Grammar subtest of the Test of Adolescent and Adult Language*

*^j^ Vocab.: Vocabulary subtest of the Wechsler Intelligence Scales for Children*

*Table 5 Results of the univariate DeFries-Fulker analysis.*

| **Group heritability estimates** | | | | |
| --- | --- | --- | --- | --- |
|  | 5% Extreme Groups^a^ | | 2.5% Extreme Groups^b^ | |
|  | N probands | Group heritability^k^ (95% confidence intervals) | N probands | Group heritability (95% confidence intervals) |
| CAST^c^ | 487 | *0.76 (0.70/0.76)* | 278 | *0.73 (0.73/1.21)* |
| CAST Social^d^ | 734 | *0.69 (0.61/0.69)* | 327 | *0.66 (0.66/1.06)* |
| CAST RRBI^e^ | 499 | *0.72 (0.58/0.72)* | 482 | *0.71 (0.57/0.71)* |
| CAST Comm.^f^ | 421 | *0.76 (0.70/0.76)* | 224 | *0.74 (0.71/0.74)* |
| FL^g^ | 569 | 0.26 (0.10/0.42) | 211 | *0.55 (0.36/0.55)* |
| MI^h^ | 284 | 0.26 (0.0.2/0.51) | 141 | 0.21 (-0.08/0.51) |
| TOAL^i^ | 258 | 0.33 (0.04/0.61) | 76 | 0.34 (-0.21/0.88) |
| Vocabulary^j^ | 284 | 0.18 (-0.05/0.41) | 180 | 0.21 (-0.05/0.48) |

*^a^ 5% extreme group: Group of participants comprising those scoring within the highest 5% of the CAST scale distributions or lowest 5% of the language scale distributions and their co-twins.*

*^b^ 2.5% extreme group: Group of participants comprising those scoring within the highest 2.5% of the CAST scale distributions or lowest 2.5% of the language scale distributions and their co-twins.*

*^c^ CAST: Childhood Autism Spectrum Test*

*^d^ CAST Social: social atypicalities subscale of the CAST*

*^e^ CAST RRBI: Repetitive, restricted behaviors and interests subscale of the CAST*

*^f^ CAST Comm.: Communication atypicalities subscale of the CAST*

*^g^ FL: Figurative Language subtest of the Test of Language Competence*

*^h^ MI: Making Inferences subtest of the Test of Language Competence*

*^i^ TOAL: Listening Grammar subtest of the Test of Adolescent and Adult Language*

*^j^ Vocabulary: Vocabulary subtest of the Wechsler Intelligence Scales for Children*

*^k^ Group heritability: indicates the extent of genetic influences on extreme scores within a defined cut-off; estimates in italics were capped at the transformed monozygotic co-twin mean*

*Table 6 Bivariate DeFries-Fulker analysis.*

| **Bivariate Heritability Estimates** | | | | | |
| --- | --- | --- | --- | --- | --- |
| Selection Variable: CAST/CAST Subscales | | | Selection Variable: Language Measures | | |
| Selection Outcome | h_2.xy_ ^a^ (5% ^b^) | h_2.xy_ (2.5% ^c^) | Selection Outcome | h_2.xy_ (5%) | h_2.xy_ (2.5%) |
| CAST^d^ – FL^e^ | -0.01/-0.13 | -0.06/-0.11 | FL – CAST | -0.02/-0.10 | -0.07/-0.10 |
| CAST – MI^f^ | -0.05/-0.15 | -0.05/-0.15 | FL – Social | -0.02/-0.06 | *-0.04/-0.02* |
| CAST – TOAL^g^ | 0.13/-0.14 | 0.22/-0.13 | FL – RRBI | -0.06/-0.03 | *-0.04/-0.02* |
| CAST – Vocabulary^h^ | *0.21/-0.15* | *0.18/-0.11* | FL – Comm. | 0.05/-0.12 | 0.03/-0.16 |
| Social^i^ – FL | 0.00/-0.09 | *-0.08/-0.06* | MI – CAST | *-0.11/-0.12* | *-0.05/-0.08* |
| Social – MI | -0.02/-0.07 | *-0.06/-0.06* | MI – Social | -0.05/-0.05 | -0.02/-0.03 |
| Social – TOAL | 0.09/-0.07 | -0.02/-0.09 | MI – RRBI | *-0.10/-0.08* | *-0.05/-0.03* |
| Social – Vocabulary | 0.16/-0.11 | 0.13/-0.10 | MI – Comm. | *-0.11/-0.13* | *-0.06/-0.10* |
| RRBI^j^ – FL | 0.09/-0.08 | 0.03/-0.08 | TOAL – CAST | *-0.08/-0.08* | *-0.05/-0.06* |
| RRBI – MI | *-0.08/-0.10* | *-0.05/-0.09* | TOAL – Social | *-0.10/-0.05* | *-0.05/-0.07* |
| RRBI – TOAL | 0.16/-0.09 | 0.10/-0.09 | TOAL – RRBI | -0.03/-0.01 | 0.06/0.07 |
| RRBI – Vocabulary | 0.08/-0.09 | 0.06/-0.08 | TOAL – Comm. | *-0.08/-0.09* | *-0.05/-0.10* |
| Comm.^k^ – FL | -0.15/-0.14 | 0.23/-0.16 | Vocab. – CAST | 0.01/-0.09 | 0.00/-0.09 |
| Comm. – MI | -0.09/-0.17 | -0.02/-0.15 | Vocab. – Social | -0.06/-0.07 | -0.05/-0.06 |
| Comm. – TOAL | 0.01/-0.13 | 0.10/-0.15 | Vocab. – RRBI | -0.03/-0.04 | 0.00/-0.03 |
| Comm. – Vocabulary | 0.14/-0.15 | *0.27/-0.17* | Vocab. – Comm. | 0.10/-0.10 | 0.04/-0.09 |
| **Genetic Correlations** | | | | | |
| Measures | 5% Extreme Groups | 2.5% Extreme Groups | Measures | 5% Extreme Groups | 2.5% Extreme Groups |
| CAST – FL | 0.03 | 0.10 | RRBI – FL | 0.17 | 0.05 |
| CAST – MI | 0.17 | 0.19 | RRBI – MI | 0.21 | 0.13 |
| CAST – TOAL | 0.20 | 0.20 | RRBI – TOAL | 0.14 | 0.16 |
| CAST – Vocab. | 0.12 | 0.00 | RRBI – Vocab. | 0.14 | 0.00 |
| Social – FL | 0.01 | 0.09 | Comm. – FL | 0.20 | 0.13 |
| Social – MI | 0.07 | 0.09 | Comm. – MI | 0.22 | 0.09 |
| Social – TOAL | 0.20 | 0.07 | Comm. – TOAL | 0.05 | 0.14 |
| Social – Vocab. | 0.28 | 0.22 | Comm. – Vocab. | 0.32 | 0.26 |

*^a^ h_2.xy_: bivariate heritability. This indicates the extent to which additive genetic influences on extreme scores on the selected measure overlap with additive genetic influences on variation in the unselected measure. Estimates are presented as the ratio of the phenotypic group correlation explained by additive genetic correlation (bivariate heritability: phenotypic group correlation). Where h_2.xy_ exceeds the phenotypic group correlation, this can be indicative of a non-additive pattern of genetic influence.*

*^b^ 5%: Extreme-scoring groups defined by scoring within the highest 5% of the CAST scales or lowest 5% of the language measures*

*^c^ 2.5%: Extreme-scoring groups defined by scoring within the highest 2.5% of the CAST scales or lowest 2.5% of the language measures.*

*^d^ CAST: Childhood Autism Spectrum Test*

*^e^ Social: Social atypicalities subscale of the CAST*

*^f^ RRBI: Repetitive, restricted behaviors and interests subscale of the CAST*

*^g^ Comm.: Communication atypicalities subscale of the CAST*

*^h^ FL: Figurative Language subtest of the Test of Language Competence*

*^i^ MI: Making Inferences subtest of the Test of Language Competence*

*^j^ TOAL: Listening Grammar subtest of the Test of Adolescent and Adult Language*

*^k^ Vocabulary: Vocabulary subtest of the Wechsler Intelligence Scales for Children*
